# Practice related factors that may impact on postpartum care for mothers and infants in Australian general practice: a cross-sectional survey

**DOI:** 10.1186/s12913-016-1508-1

**Published:** 2016-07-11

**Authors:** Wendy E. Brodribb, Benjamin L. Mitchell, Mieke L. van Driel

**Affiliations:** Discipline of General Practice, The University of Queensland, Health Sciences Building, Herston, Queensland 4029 Australia

**Keywords:** Postpartum, Postnatal, Primary care, General practice, Practice nurse, Australia

## Abstract

**Background:**

While there is a significant focus on the health and well-being of women during pregnancy, labour and birth, much less emphasis is placed on the care of postpartum women and their infants in primary care following the birth. Some studies have investigated the role of GPs in postpartum care, and others examined facilitators and barriers to mothers accessing care. However there is little information available to investigate the effect of practice related factors on access to care of mothers and infants at this time.

**Methods:**

A 20-item questionnaire for completion by the practice managers was mailed to 497 general practices in Southern Queensland, Australia between February and July 2013. Questionnaire items included practice demographics, practice procedures and personnel including appointment scheduling, billing, practice nurse function and qualifications and a free-text option for comments. Descriptive statistics are presented as numbers and percentages. Chi Squared test compared practice location with methods of identification of postpartum women, practice size with other Queensland data and ANOVA compared practice size with the number of postpartum appointments. Logistic regression was used to predict variables that were related to booked appointment times. Free text responses were grouped in common themes.

**Results:**

The response rate was 27.4 %. At 67.2 % of the practices, mothers had to self-identify as needing a postpartum consultation and most consultations were allocated 15 minutes or less. Only 20 % of practices accepted the government insurance payment (bulk-billing) for all maternal and infant services, with more practices bulk-billing children only. Out-of-pocket expenses ranged from $10-$60. Nearly 80 % of practice nurses saw postpartum mothers or infants ‘nearly always’ or ‘sometimes’. Approximately 30 % had midwifery or child health training. There were higher odds of longer booked appointment times for solo practitioner practices (unadj OR 3.30 95%CI 1.03-10.57), but no other variables predicted booked appointment times

**Conclusions:**

This study identified a number of practice related factors that, if addressed, could positively impact on postpartum care. These include ensuring ongoing practice relationships to assist with booking appropriate consultation times and guaranteeing that there are no financial impediments to women accessing care. Some factors can easily be adapted within practices. Others would require changes of policy at a local or national level.

**Electronic supplementary material:**

The online version of this article (doi:10.1186/s12913-016-1508-1) contains supplementary material, which is available to authorized users.

## Background

Around 300,000 infants are born in Australia each year: the vast majority within the hospital sector [1]. While there is significant focus on the health and well-being of the mother and infant during the pregnancy and intrapartum period (including the postpartum hospital stay) few studies examine methods to improve post-discharge primary care services, especially in general practice, for postpartum women and infants.

A strong primary care system that incorporates continuity of care with an ongoing doctor-patient relationship [2] and coordinated and comprehensive services has the capacity to reduce health costs and results in better population health outcomes [3]. Patients are more likely to engage with primary care and general practice if their health care provider is familiar to them, is thorough with physical examinations and asks about social and emotional issues [4]. The ease of making appointments and limited waiting times once at the surgery are also important [4, 5]. Another significant factor influencing access to health care is affordability, especially when it relates to preventative care [6].

While these factors may also be pertinent in the postpartum period, there is little research in developed countries specifically targeting access to primary care for healthy postpartum women and infants. Continuity of care and connection with practice staff, especially involvement in antenatal care, are facilitators for women to access postpartum care [7–9], as are reminders sent from the surgery [9]. However, younger women and those on lower incomes or who are socially disadvantaged are less likely to attend postpartum consultations [7–9]. If the health care focus is primarily on the infant, women may also not understand the importance of a postpartum check for themselves, especially if they are feeling well [7, 10].

While health services vary throughout the world, general practitioners (GPs) in Australia provide a continuum of care from prepregnancy through to the postpartum period and beyond, are the main health care providers for women, infants and children and are the gatekeepers for all specialist care. In a recent Queensland study 87.8 % of women first visited a GP when they thought they may have been pregnant and a quarter of these women chose GP shared antenatal care where the GP undertakes most antenatal visits, but hospital obstetric and midwifery services are responsible for care during labour, birth and the immediate postpartum period [11]. Women may also leave general practice and access a midwifery model of care, public hospital obstetric care and private obstetric care for their pregnancy and birth. In Queensland, women who birth in free public sector hospitals (72 % of all women) [1] are encouraged to return to their GP for routine postpartum care within the first 2 weeks after birth and at 6 weeks to assess their health and wellbeing and that of their infant and to manage any issues that arise. A small proportion of women will continue with midwifery care until 6 weeks. It is assumed that women who birth in the private sector return to see their obstetrician and paediatrician around 6 weeks postpartum, but many will visit their GP earlier than that. Most will not have further contact with specialist care until the next pregnancy. Overall, approximately 45 % of women visit their GP for themselves or their infant within seven days of hospital discharge and 88 % do so by three months. This is much higher than the proportion of women who visit a free Child and Family Health Clinic by three months (66 %) [11] and quite different to some other Australian states where most women have contact with Child and Family Health Clinics in the early postpartum weeks [12].

Even though general practitioners are integral players in postpartum care, approximately 40 % of GPs recently surveyed in Southern Queensland were uncertain or thought that women were not happy with the way postpartum care was provided in general practice [13]. Consistency in recommendations for when women should see their GP, increasing the length of consultations to ensure that the health and wellbeing of both mother and infant are addressed and improving the skill and expertise of the GP have been identified as particular issues to address [13, 14]. However, GPs work within a system and factors that facilitate or are barriers to women accessing and receiving postpartum care also need to be explored. Some of these factors may relate to connection to the practice, appointment scheduling, continuity of carer and out-of-pocket expenses. It may also include the use of other health care providers such as practice nurses (PNs - registered nurses who work within general practice) to provide some of the services. Previous studies have focused on mothers’ experiences, particularly of those who do not return for postpartum care [7–10], but no previous research has explored these issues from a practice perspective.

Therefore, the aim of this paper is to investigate practice-based factors that may impact on the access to routine care for postpartum mothers and their infants within general practice.

## Method

### Participants

Participants were general practices in the catchment of the Metro North Brisbane and Darling Downs and South West Queensland Medicare Locals (government sponsored organisations to support primary health care providers) or associated with the University of Queensland, Discipline of General Practice and based in Southern Queensland.

### Procedure

As there is no central register of general practices, contact details were obtained from one Medicare Local, the telephone book, internet sites and from personal contacts to develop a comprehensive list of active general practices in the geographic area of interest. Between February and July 2013, 497 general practices were contacted by phone and mailed or faxed a questionnaire and participant information sheet for the Practice Manager or similar staff member to complete. A follow-up phone call was made two weeks later if there had been no response. The questionnaires could be completed in hard copy and returned by fax or in a reply paid envelope or completed online. Ethics approval was obtained from the Behavioural and Social Sciences Ethical Review Committee of the University of Queensland (Approval no 2012001264). Return of the questionnaire was deemed to be informed consent.

### Measures

A 20 item questionnaire (Additional file 1) developed for this study was reviewed by four GPs, a Child and Family Health nurse, an academic with an interest in maternal and infant health and a community representative. Items included: general practice location and the number of GPs in each practice; an estimate of the number of routine postpartum appointments (for mother or infant) made in the previous fortnight; the length of time usually booked for routine appointments for mothers and for infants in minutes (transformed into a categorical variable ≤15 mins, >15 mins); qualifications of PNs, and how often each PN saw postpartum mothers and infants (nearly always, sometimes, rarely or never); how practices identified new mothers and infants (All patients are asked why they need an appointment when they ring, Staff are aware of the patients of the practice who are pregnant and are asked if they have had their baby, We rely on the mother to inform the person making the appointment); which patients are bulk-billed (consultations charged directly to the government through Medicare [a universal health insurance scheme] with no gap fee for the patient) and what out-of-pocket expenses mothers could expect. There was also a free-text option for further comments.

### Data analysis

Analysis was undertaken using SPSS for Windows (version 21.0) [15]. Descriptive statistics are presented as numbers and percentages. Chi Squared test compared practice location with how postpartum women were identified and the number of GPs in the practices with other Queensland data. The association between the number of GPs in the practices and mean number of postpartum consultations in the previous fortnight was assessed using ANOVA. To find variables that are associated with appointment booking times logistic regression was used to determine unadjusted Odds Ratios and 95 % confidence intervals. Variables were also assessed for their independent association with booked appointment time. Free text responses were grouped around common threads.

## Results

Completed questionnaires were received from 136 practices (response rate 27.4 %). Practice location and the number of GPs in the practices can be found in Table 1. Compared to a representative sample of practices in Queensland [16], participant practices were more likely to have 6 or more GPs. Only 12.7 % of practices did not have a male GP, while 15 % had no female GPs. All but one practice had at least one doctor undertaking shared antenatal care and GP obstetricians were available in 20 % of practices.

**Table 1:** GP practice demographics

		study practices % (*n*)	Representative GP practices in Queensland % (*n*)	Chi Squared
Location of practice (*n* = 136)^a^				
	Metro Brisbane	48.5 (66)		
	Regional or provincial city	23.5 (32)		
	Country town	28.0 (38)		
Number of GPs in practices (*n* = 134)				
	1	14.2 (19)	25 (77)	χ^2^(2) 56.45 *p* < .0001
	2-5	51.5 (69)	58.4 (180)	
	6 or more	34.3 (46)	16.5 (51)	

^a^Metro Brisbane – Greater metropolitan Brisbane; regional or provincial city - > 15,000 people; country town - ≤ 15,000 people

The mean number of postpartum consultations (for mother or infant) in the previous fortnight per practice, analysed by practice size is found in Table 2. As would be expected, the larger the GP practice, the more postpartum consultations undertaken.

**Table 2:** Mean number of postpartum appointments in the previous fortnight for size of practice

Number of GPs in practices	Mean no of appointments (95 % CI)	ANOVA
1	2.83 (1.57-4.10)	F = 14.17. *p* < .0001
2-5	5.74 (4.35-7.12)	
6 or more	12.21 (8.73-15.68)	

The majority of all consultations were allocated 15 minutes or less (See Table 3). Compared to practices with six or more GPs, practices with only one GP had more than three times higher odds of booking consultations for more than 15 minutes for mothers, but not infants (See Table 4).

**Table 3:** Booked appointment times, methods of identification and billing practices that may impact on postpartum care

Variable	% (*n*)
Booked appointment time for mothers (*n* = 126)	
≤15 minutes	59.5 (75)
>15 minutes	40.5 (51)
Booked appointment time for infants (*n* = 126)	
≤15 minutes	54.8 (69)
>15 minutes	45.2 (57)
Method of identification for postpartum consultation (*n* = 133)	
All patients asked why they need appointment	22.9 (30)
Staff aware of pregnant patients	38.2 (50)
Rely on mothers to inform staff if need to postpartum appointment	67.2 (88)
Practice billing (*n* = 133)	
Universally bulk-bill	19.5 (26)
Bulk-bill children	54.9 (73)
Bulk-bill infants or mothers at doctors’ discretion	69.2 (92)
Cost of consultation for mother or infant (*N* = 122)	
No charge	32.8 (40)
$1-20	12.3 (15)
$21-30	37.7 (46)
>$30	17.2 (21)

**Table 4:** Practice characteristics and univariate associations between characteristics and appointment booking time of > 15 minutes for infants and mothers

		Infant appointment booked time		Mother appointment booked time	
		>15 minutes^a^		>15 minutes^a^	
		% (*n*)	Unadjusted OR (95 % confidence interval)	% (*n*)	Unadjusted OR (95 % confidence interval)
Location of practice^b^					
	Metro Brisbane	50.9 (29)	1	51.0 (26)	1
	Regional or provincial city	21.1 (12)	.68 (.28-1.63)	13.7 (7)	.38 (.14-1.02)
	Country town	28.1 (16)	.90 (.39-2.08)	35.3 (18)	1.39 (.60-3.20)
Number of GPs in practices (*n* = 134)					
	1	17.9 (10)	2.0 (.63-6.2)	20.0 (10)	3.30 (1.03-10.57)*
	2-5	50.0 (28))	1.08 (.49-2.36)	54.0 (27 )	1.68 (.74-3.82)
	6 or more	32.1 (18)	1	26.0 (13)	1
PN to see mothers and infants (*n* = 135)					
	Yes	80.4 (45)	.70 (.27-1.78)	80.4 (41)	.72 (.28-1.84)
	No	19.6 (11)	1	19.6 (10)	1
All patients asked why they need appointment (*n* = 131)					
	Yes	30.4 (17)	1.88 (.82-4.31)	30.0 (15)	1.71 (.75-3.92)
	No	69.6 (39)	1	70.0 (35)	1
Staff aware of pregnant patients (*n* =131)					
	Yes	39.3 (22)	1.14 (.55-2.36)	46.0 (23)	1.81 (.87-3.79)
	No	60.7 (34)	1	54.0 (27)	1
Rely of mothers to inform staff if need to postpartum appointment (*n* = 131)					
	Yes	66.1 (37)	.91 (.43-1.93)	62.0 (31)	.68 (.32-1.44)
	No	33.9 (19)	1	38.0 (19)	1
Cost of consultation (*n* = 122)					
	No charge	33.3 (17)	1	38.6 (17)	1
	$1-$20	13.7 (7)	.93 (.28-3.11)	15.9 (7)	.93 (.28-3.11)
	$20-$30	37.3 (19)	.88 (.36-2.15)	34.1 (15)	.59 (.34-1.47)
	>$30	15.7 (8)	.65 (.22-1.96)	11.4 (5)	.33 (.10-1.10)

^a^compared to ≤15 minutes

^b^Metro Brisbane – Greater metropolitan Brisbane; regional or provincial city - > 15,000 people; country town - ≤ 15,000 people

*p < .05

At 67.2 % of the practices mothers had to self-identify that they were attending for a postpartum consultation when making an appointment (Table 3). Practices in country towns were more likely to be aware of women who may have given birth (56.8 %) compared to practices in metropolitan areas (32.3 %) or in regional/provincial cities (28.1 %) (χ^2^(2) = 7.7 *p* = .02). However, method of identification did not affect length of booked appointment for the mother or infant (See Table 4).

Billing practices can be found in Table 3. The usual out-of-pocket charge in practices that did not bulk-bill or charged more than the government rebate ranged between $10 and $60 with 38 % of practices charging between $20 and $30. Out-of-pocket expenses were not related to booked appointment times (See Table 4). Free text comments suggested that infants who did not have a Medicare number (a universal health insurance number which enables bulk-billing) created billing problems for the practice as the infant could not be bulk-billed.

Nearly 90 % of practices employed at least one PN and PNs saw mothers or infants in 79.4 % of all practices. Of the 279 PNs identified in the study, two thirds (63.8 %) would ‘nearly always’ and 20.2 % would ‘sometimes’ see a mother and infant in the first 8 weeks postpartum. Only 29.8 % of the PNs had qualifications related to maternal and infant health (see Table 5). Length of the booked appointments was not significantly different between practices in which PN saw mothers and infants and those that did not (see Table 4).

**Table 5:** Practice nurse characteristics

Variable	% (*n*)
Number of PNs in practices (*n* = 136)	
0	10.4 (14)
1	22.2 (30)
2	33.3 (45)
3	10.4 (14)
4 or more	23.9 (32)
Frequency of PNs seeing mothers and infants in the first 8 weeks postpartum (*n* = 279)	
Nearly always	62.8 (175)
Sometimes	20.2 (56)
Rarely	7.6 (21)
Never	9.4 (26)
PN qualifications relevant to maternal and child health (*n* = 275)	
None	70.2 (193)
Midwifery	18.9 (52)
Child health	8.4 (23)
Other	6.2 (17)

## Discussion

This is the first study to investigate practice related factors that may affect access to routine postpartum care in general practice. A high proportion of the practices involved in the study undertook antenatal shared care and all provided community postpartum care for mothers and infants on a regular basis.

The majority of consultations were allocated 15 minutes or less with solo GP practices having significantly higher odds of longer consultations for mothers. While quality of postpartum care cannot be assessed by consultation time alone, previous studies have found that GP consultations for either the mother or infant often take longer than 15 minutes [13], and take longer than anticipated [14]. It has also been found that short and rushed consultations were an impediment to women seeking care and to discussing issues of concern with the GP [14]. This problem is accentuated if an appointment is only made for either the mother or the infant, although both mother and infant need to be seen. More broadly, there is evidence that in Australia the number of long consultations for children has decreased over recent years [17], although the proportion of consultations for children has remained static [18]. This may be in response to the emphasis on chronic disease management in general practice [17]. Similar studies have not yet been undertaken focusing on postpartum consultations in general, or for postpartum women in particular.

While routinely making double or long appointments for women and their infants, or ensuring an appointment is made for both mother and infant, has been suggested to overcome inadequate consultation times [19], this strategy would only be effective if the practice staff were aware of the purpose of the appointment. In this study there was no difference in consultation times with methods of identification of postpartum mothers. However, we were unable to ascertain if timely identification resulted in two appointments being made rather than one. Of the practices surveyed, 67 % relied on mothers to inform them that they had recently given birth and would thus require extra time for mother and infant. In country towns with smaller populations, practice staff were more likely to be aware of pregnant patients and proactively asked whether the birth had occurred. Not only does this allow for appropriate appointment scheduling, but also reinforces the connection with the practice that facilitates women accessing care [9]. Strategies such as giving information to all pregnant women from the practice, regardless of the model of maternity care, about services offered postpartum and the importance of postpartum follow-up may also be advantageous when staff are less familiar with the patient population.

As noted previously, cost is a significant factor in people’s willingness to access to health care in general [6] and may have a negative impact on women accessing care in the postpartum period within general practice. In this study the majority (54.5 %) of practices automatically bulk-billed children, but in nearly 70 % of practices, families had some out-of-pocket expenses for either the infant or mother. In some situations this out-of-pocket expense was as high as $60 per consultation. Problems concerning payment for consultations for infants before they received a Medicare number were mentioned in the free text section of the questionnaire, and mothers may be required to pay and then claim from Medicare in these situations. While each practice can determine who it charges and how much, mothers are vulnerable at this time and should not be discouraged to seek care if needed, by financial impediments. This is especially important for mothers from a lower socio-economic background who tend to have more complex health care needs and are less likely to seek postpartum care [7]. We were unable to correlate cost with area of practice. However, a recent study found that cost of a GP visit for an infant under 12 months was lower for families if the mother had a lower level of education and the family had a lower level of income. In contrast to a previous study that found patients from low socio-economic status areas were less likely to receive longer GP consultations [20] the study by Golenko et al. [21]. found that income was not related to the number or length of consultations. These findings suggest that families from lower socio-economic settings were able to receive comparable free or low cost care to other families [22].

One way to improve practice efficiency and patient access to comprehensive care, especially if the appointment time with the GP is short, is to use PNs to undertake some routine or preventative consultations. In this study in approximately 80 % of GP practices a PN ‘nearly always’ or ‘sometimes’ saw a mother and/or infant within the first eight weeks postpartum. However, only 19 % of the nurses held midwifery qualifications, and only 8 % held child health qualifications. These figures are slightly better than the PN population in general where 10.9 % have midwifery qualifications [23]. It appears that many nurses rely on personal experience or guidance from their supervising GP (who may not have the requisite knowledge) to develop the necessary skills to undertake these consultations. Most research conducted with PNs has focused on chronic disease management with the few studies reporting on PN involvement in postpartum care finding they often lack the knowledge and skills to adequately care for the women and infants they see [24, 25].

Nevertheless the Australian Primary Health Care Nurses Association’s (APNA) website indicates that the PN role includes well baby and toddler care, maternal postpartum care and parenting advice and referral for specialised service if indicated [26]. The one randomised controlled trial where PNs were trained to deliver a motivational interviewing intervention about breastfeeding at the regular two month immunisation visit, found an improvement in exclusive breastfeeding rates before six months [27]. PNs therefore, can play an important role in improving health outcomes for mothers and their babies as long as they have adequate education, which seems to be lacking at present.

### Limitations

Although the study response rate was less than 30 % this is not uncommon in studies of general practices [28]. The fact that the practices were from metropolitan as well as regional and rural/remote areas and that a range of practice sizes were included increases the generalisabilty of the results. However, the data may be indicative of practices with an interest in maternity care as nearly all had at least one GP who conducted shared antenatal care and in 20 % of practices there was a GP obstetrician. These findings are therefore likely to be a ‘best case’ scenario. All data are self-reported and have not been verified by audit or by cross-checking with patients.

## Conclusions

While there are data about why some women do not access postpartum primary care, little is known about factors that can impact on timely care of postpartum women and infants from a general practice perspective. This study has identified a number of practice related issues that could be addressed to improve general practice postpartum care. For example, strategies to ensure connection of mothers to the general practice and continuity of practice relationships would assist in booking appropriate appointments. Allocating time to be seen by a PN may also be possible if postpartum mothers and infants are identified when an appointment is made. Providing opportunities for PNs to upskill in the management of routine postpartum care for the mother and the infant could improve efficiency from a practice perspective and enhance outcomes for mother and child. Finally, costs to mothers for attendance at general practice for postpartum care must be considered.

Some factors can be easily adapted in the current Australian GP practice environment, such as scheduling and PN involvement. Other factors may need to be reviewed at a local policy level (e.g. communication and transition from hospital to primary care) or a national policy level (e.g. remuneration for the more time-intensive postpartum consultation).

## Abbreviations

GP, general practitioner; PN, practice nurse.

## Additional file

Additional file 1: POSTPARTUM CARE IN GENERAL PRACTICE – What happens and can it be improved?. (DOCX 25 kb)
