# Supplementary material for: Practice related factors that may impact on postpartum care for mothers and infants in Australian general practice: a cross-sectional survey
Source: BMC Health Serv Res. 2016 Jul 11;16:244. doi: 10.1186/s12913-016-1508-1 (PMC4940844; doi:10.1186/s12913-016-1508-1)
Supplement: Additional file 1: — POSTPARTUM CARE IN GENERAL PRACTICE – What happens and can it be improved?. (DOCX 25 kb) [file 12913_2016_1508_MOESM1_ESM.docx]

**POSTPARTUM CARE IN GENERAL PRACTICE –
What happens and can it be improved?**

Thank you for completing this questionnaire about your practice and how mothers and babies are cared for in the FIRST 8 WEEKS POSTPARTUM. The information obtained from your responses will help us develop an understanding about how mothers and babies in the postpartum period are managed in general practice. We plan to develop guidelines and investigate ways of improving maternal and infant care.

To complete the questionnaire please tick the appropriate box, or complete the question in the space provided. The questionnaire will take approximately 5 minutes to complete. Unless otherwise indicated please give only one response for each question.

Please return the questionnaire by fax (07 33465178), in the reply paid envelope, or post to Dr Wendy Brodribb, Discipline of General Practice, Level 8 Health Sciences Building, Herston 4029. You may also complete the questionnaire online at <https://www.surveymonkey.com/s/FKMTS92> using the identification code above.

1. What is the postcode of your practice? ______
2. Where is your practice located?

☐ Brisbane metropolitan

☐ Regional city (> 50,000)

☐ Provincial city (15001 – 50,000)

☐ Large country town (6001-15000)

☐ Medium sized country town (2001-6000)

☐ Small country town (<2000)

How many doctors usually work in the practice?

|  | 1. **Male** | 1. **Female** |
| --- | --- | --- |
| Part-time |  |  |
| Full-time |  |  |
| Full time equivalents |  |  |

1. How many of these doctors do shared antenatal care? ______
2. How many of these doctors are GP obstetricians? ________
3. How many practice nurses does the practice employ? (For the purpose of this study ‘practice nurse’ also includes midwives, physician assistants or equivalent)

☐ None (Go to Question 9)

☐ One

☐ Two

☐ Three

☐ More than three

1. How commonly would a practice nurse see a mother and baby who comes to the practice in the first 8 weeks postpartum?

|  | **Nurse 1** | **Nurse 2** | **Nurse 3** | **Nurse 4** |
| --- | --- | --- | --- | --- |
| Nearly always | ☐ | ☐ | ☐ | ☐ |
| Sometimes | ☐ | ☐ | ☐ | ☐ |
| Rarely | ☐ | ☐ | ☐ | ☐ |
| Never | ☐ | ☐ | ☐ | ☐ |

1. What qualifications related to maternal and child health does the practice nurse/s have?

|  | **Nurse 1** | **Nurse 2** | **Nurse 3** | **Nurse 4** |
| --- | --- | --- | --- | --- |
| None | ☐ | ☐ | ☐ | ☐ |
| Midwifery | ☐ | ☐ | ☐ | ☐ |
| Child Health | ☐ | ☐ | ☐ | ☐ |
| Health worker | ☐ | ☐ | ☐ | ☐ |
| IBCLC (lactation consultant) | ☐ | ☐ | ☐ | ☐ |
| Other | ☐ | ☐ | ☐ | ☐ |

1. Are there any other health care professionals attached to the practice (eg lactation consultants, midwives, physiotherapists, dietitians) who are available to see mothers and their babies if problems are identified?

☐ No (go to Question 12)

☐ Yes – please explain________________________________________________________

­­­­­­­­­­­­­­­­___________________________________________________________________________

1. Are mothers able to access these services easily (timely appointments and cost)?

☐ Yes

☐ No – please explain________________________________________________________

­­­­­­­­­­­­­­­­___________________________________________________________________________

1. Is the practice located adjacent to or within walking distance of

|  | **Yes** | **No** |
| --- | --- | --- |
| A pharmacy that has a nurse who runs a baby clinic | ☐ | ☐ |
| A Queensland Health run Child and Family Health clinic | ☐ | ☐ |

1. Please estimate how many appointments for routine postnatal and neonatal checks have been made for women and babies in the last fortnight? ­­­­­­­­­­­­­­_________

For what length of time is a mother and/or baby usually booked for a routine postnatal/neonatal visit in your practice?

|  | 1. **Time booked (mins) within 2 weeks postpartum** | 1. **Time booked (mins) 3-8 weeks postpartum** |
| --- | --- | --- |
| Mother |  |  |
| Baby |  |  |

1. How are mothers/babies who need a routine postnatal/neonatal check identified when they ring to make an appointment?

☐ All patients are asked why they need an appointment when they ring

☐ Staff are aware of the patients of the practice who were pregnant and are asked if they have had their baby

☐ We rely on the mother to inform the person making the appointment

☐ Other (please explain)______________________________________________________

____________________________________________________________________________

1. In your practice do you (please mark all that apply)

☐ Universally bulkbill

☐ Bulkbill health care card holders

☐ Bulkbill children

☐ Bulkbill immunisations

☐ Bulkbill antenatal care

☐ Private fee (bulkbill at doctor’s discretion)

☐ Other (please explain) __________________________________________________________

_______________________________________________________________________________

1. What ‘gap’ does a mother usually have to pay for a postpartum consultation for herself and/or her baby? ­­­­­­­­­­­­­­­­­­­­­­­­­­­____________________________________________________
2. What item number is usually charge for a routine postpartum visit in your practice for a new mother?__________
3. What item number is usually charge for a routine neonatal visit in your practice for a new baby?­­­­­­­­­­­­­­­­­­­­_____________

Is there any other information you would like to add?

**THANK YOU VERY MUCH FOR YOUR ASSISTANCE WITH THIS RESEARCH PROJECT**
